# Cannabidiol versus placebo as adjunctive treatment in early psychosis: study protocol for randomized controlled trial

**DOI:** 10.1186/s13063-023-07789-w

**Published:** 2023-11-30

**Authors:** T. Dixon, K. S. Cadenhead

**Affiliations:** Department of Psychiatry, University of California San Diego, 9500 Gilman Drive 0810, La Jolla, CA 92093-0810 USA

**Keywords:** Psychosis, Schizophrenia, Cannabidiol, Attenuated psychosis syndrome, Stress, Inflammation, Hyperphagia, Metabolism

## Abstract

**Background:**

Psychotic disorders are a leading cause of disability in young adults. Antipsychotics have been the primary intervention for psychosis for over 60 years, and yet, we have made little progress in treating negative symptoms, neurocognition, and functional disability. There is growing evidence that cannabidiol (CBD) is effective in treating positive psychotic symptoms, possibly also negative and neurocognitive symptoms, and moreover is well tolerated compared to other psychotropic medications. Anecdotally, patients participating in the Cognitive Assessment and Risk Evaluation (CARE) Early Psychosis Treatment Program at the University of California, San Diego, are self-administering CBD and report subjective improvement in stress, anxiety, and ability to cope with symptoms. The overarching aim of the trial is to explore the effectiveness of CBD augmentation on symptoms and neurocognition in early psychosis while also exploring the mechanism of action of CBD and predictors of response to treatment. The mechanism by which cannabidiol has a therapeutic effect on psychosis is poorly understood. Recent evidence has suggested that CBD may reduce stress and pro-inflammatory biomarker levels. Endocannabinoids also have powerful roles in eating behavior, reward, and mood, indicating these neurotransmitters may play a role in reducing hyperphagia and metabolic abnormalities that are present early in the course of psychotic illness and exacerbated by antipsychotic medication. The neurophysiological effects of CBD have been studied in animal models of psychosis that show improvements in information processing in response to CBD, but there are no studies in individuals with early psychosis.

**Method:**

A total of 120 individuals in the early stages of psychosis will be randomized to 1000 mg of CBD versus placebo as an adjunct to existing treatment in a 8-week, double-blind superiority randomized control trial. The primary outcome measures are symptoms and neurocognition.

**Discussion:**

We hypothesized that CBD will improve symptoms and neurocognition as well as secondary outcome measures of neurohormones, inflammation, eating behaviors, and information processing. Importantly, predictors, moderators, and mediators of the CBD effects will be examined. A better understanding of which individuals are likely to respond to CBD can inform treatment planning and personalize treatment.

**Trial registration:**

ClinicalTrials.gov NCT04411225. Registered on June 2, 2020.

## Introduction

The epidemiological literature demonstrates an association between the early use of cannabis, greater lifetime use, more potent strains, and later risk for psychotic illness [1–4]. Cannabis use during adolescence is now considered a significant environmental factor that is not only associated but might contribute to the development of psychotic disorders [5]. The prefrontal cortex (PFC), with a key role in the pathophysiology of psychosis, continues to develop throughout adolescence and dynamic fluctuations in components of the endocannabinoid (eCB) system also occur throughout adolescent development [6–8]. The critical role of the eCB system in neuronal development and synaptic plasticity [9] suggests that this eCB-controlled regulation relates to the fine-tuning of PFC circuits established during adolescence. Translational studies have revealed the role of eCB receptors and endocannabinoids in dopamine and glutamatergic regulation, both of which are linked to the pathophysiology of schizophrenia [10–13].

Schizophrenia and related psychotic disorders are neurodevelopmental disorders that fully emerge during late adolescence or early adulthood and are a leading cause of morbidity, mortality, and disability. Over the last 2 decades, the scientific focus in psychosis research has shifted to early identification and prevention with the prospect of preventing neuropathological changes and the devastating effects of this disabling illness. Patients who meet the criteria for attenuated psychosis syndrome (APS) have up to a 30% risk of conversion to a full psychotic syndrome within 2 years [14], yet effective pharmacologic interventions, which can disrupt the progression of symptoms, have yet to be identified. Individuals who have already progressed to the early stages of psychosis respond to antipsychotic medication in terms of their positive psychotic symptoms but the negative and neurocognitive symptoms are very difficult to treat, leading to long-term difficulties in social, role, and global functioning and often lifelong disability. In addition, the metabolic abnormalities that often accompany psychotic illness lead to chronic medical issues.

Because cannabidiol (CBD) can reverse many of the biochemical, physiological, and behavioral effects of CB1 receptor agonists such as tetrahydrocannabinol (THC), a handful of studies have explored the possible role of cannabinoids, including CBD, in the treatment of psychosis. There is growing evidence that CBD has the potential to improve positive, negative, and cognitive symptoms of psychosis [15, 16], but the mechanism of action is not well understood and the efficacy of CBD has not been studied in EP when it is likely to have the most robust effect given the greater neuroplasticity in the early stages of illness.

## Previous CBD trials in psychotic illness: symptoms and side effects

There are relatively few studies of CBD alone or as augmentation in psychotic illness. Leweke et al. [17] compared cannabidiol versus amisulpride 200–800 mg per day each in a 4-week double-blind, parallel-group, randomized, active-controlled clinical trial of 42 patients. Treatment with CBD versus the antipsychotic was associated with significantly fewer extrapyramidal symptoms, less weight gain, and lower prolactin increase. CBD was well tolerated and did not affect hepatic or cardiac functions. There was a significant clinical improvement assessed over 28 days as measured by a decrease in the Positive and Negative Syndrome Scale (PANSS) among both amisulpride and CBD groups. In another study, McGuire et al. [16] performed a double-blind, parallel-group trial of adjunctive CBD (1000 mg/day) or placebo that was added to existing antipsychotic treatment in 88 patients with schizophrenia. CBD was well tolerated, and rates of adverse events were similar in the CBD (30 AEs in 15 patients) and placebo group (35 AEs in 16 patients). Patients in the CBD group compared to the placebo group had lower positive psychotic symptoms and were more likely to be clinician-rated as improved and as not severely unwell. Gastrointestinal events (nausea and diarrhea) were the most common in the group taking CBD and were reported as mild, resolving without intervention. Only two events in the CBD group were considered treatment related (dyslipidemia and nausea), and both were considered mild. Boggs et al. [18] performed a 6-week, randomized, placebo-controlled, parallel group, fixed-dose study of CBD (600 mg/day), or placebo augmentation in 36 stable antipsychotic-treated patients with chronic schizophrenia for tolerability of CBD treatment and cognitive effects. Side effects were similar between CBD and placebo except sedation was more prevalent in the CBD group. One participant in the CBD arm withdrew early due to sedation. Approximately 20% of the CBD participants reported mild sedation and 5% in the placebo arm reported sedation. The most common side effects noted were gastrointestinal in nature, but these were more frequent in the placebo arm 10/18 (51%) vs 6/18 (33%) on CBD and not statistically different. There was again an overall significant decrease in PANSS ratings among study subjects without reaching significance for correlation with the CBD group (drug × time interaction), and there was no improvement in cognitive scores in any group [18]. Notably, one open-label pilot study [19] evaluated CBD administration in addition to usual therapy for six patients with diagnoses of Parkinson’s disease and psychosis over 6 months. CBD did not worsen motor function or cause adverse effects, and there was a significant improvement in symptoms per the Brief Psychiatric Rating Scale and Parkinson Psychosis Questionnaire. Adjacent and related to psychosis, a recent trial randomized adults above 60 years of age with major neurocognitive disorder with behavioral disturbances to receive broad-spectrum cannabis oil, and it was found that patients experience a decrease in the Cohen-Mansfield Agitation Inventory score by week 16 [20]. CBD effects in early psychosis patients, where there is the greatest potential for neuroprotection and prevention of deterioration, have yet to be studied.

## Mechanism of action of CBD

There are a number of hypothesized mechanisms by which CBD might exert its therapeutic benefit. Translational studies have revealed the role of cannabinoid (CB) receptors and endocannabinoids in dopamine and glutamatergic regulation, immune function, energy metabolism, and the pathophysiology of schizophrenia [10–13]. CBD does not bind to the known cannabinoid receptors and its mechanism of action remains unknown [21]. Several provisional theories exist including CBD facilitates endocannabinoid signaling by inhibiting the cellular uptake and enzymatic hydrolysis of endocannabinoids [22]. Alternative mechanisms have been postulated; it binds to serotonergic (5HT1A) receptors, inhibits adenosine uptake, and can activate vanilloid (TRPV1) receptors at micromolar concentrations [22–24]. Regardless of the mechanism, CBD is not psychoactive. In fact, it attenuates some of the psychoactive effects of THC [25, 26].

In clinical studies, anandamide, an endogenous CB1 receptor agonist, has been shown to be elevated in antipsychotic- and cannabis-naïve patients with schizophrenia [27, 28]. Koethe et al. [10] have proposed a model of psychosis in that the endogenous agonists like anandamide may rise in response to increased dopamine transmission and provide neuroprotection. Anandamide reuptake and hydrolysis are inhibited by cannabidiol, the second most abundant component of *Cannabis sativa* (besides THC), which has weak partial antagonistic properties at the CB1 receptor. Studies in animals, healthy humans, and patients with schizophrenia suggest that cannabinoids have a pharmacologic profile similar to antipsychotic drugs [29].

CBD is rapidly distributed into the tissues with a high volume of distribution of ~ 32 L/kg [30]. Due to its high lipophilicity, it tends to accumulate in adipose tissues. It is highly bound to plasma proteins and circulating blood cells. CBD undergoes CYP3A- and CYP2C-dependent phase I metabolism to 7-hydroxy-CBD, which is further metabolized and excreted, in feces and to a lesser extent in urine. CBD has an estimated terminal half-life of 18–32 h and a clearance of 57.6–93.6 L/h [31]. CBD is metabolized mainly by the cytochrome P450 (CYP) 2C19 and CYP3A4 isoenzymes, which are induced by several anti-epileptic drugs (e.g., carbamazepine, topiramate, phenytoin) and are inhibited by others (e.g., valproate). CBD can also inhibit the CYP2C family of isozymes (inhibition constant [Ki] = 1–10 μM) and CYP3A4 (Ki = 1 μM) [32].

In a recent study evaluating pharmacokinetics and safety profiles in children treated with CBD for intractable epilepsy, 34 patients ages 4–10 years with treatment-resistant epilepsy due to Dravet syndrome were treated with CBD at doses from 5 to 20 mg/kg/day in two divided doses [33]. Pharmacokinetic studies were conducted at 2–3 and 4–6 h post-dose. Plasma concentrations of plasma concentrations of CBD, 6-OH-CBD, and 7COOH-CBD were determined. Peak levels of CBD and other metabolites examined occurred around 2 ½ h post-administration. At all doses and time points, 7-COOH-CBD was the most abundant circulating metabolite while concentrations of 6OH-CBD were consistently < 10%.

## CBD and neurocognition

Studies of the neurocognitive effects of CBD in humans have shown mixed results in chronic psychosis. In a follow-up to their original CBD/Amisulpride study, Leweke et al. [34] observed cognitive improvements in both the CBD and amisulpride groups but in different domains, in an anandamide-independent fashion. The CBD group had improved sustained attention and visuomotor coordination and the amisulpride group had enhanced working memory performance [34]. Hallak and colleagues [35] assessed patients with schizophrenia on the Stroop Color Word test following acute administration of 300 or 600 mg of CBD and did not find any beneficial effects with single dosing [35]. Furthermore, there have been two studies looking at add-on therapy, Boggs et al. [18] added 600 mg of CBD daily for 6 weeks in 36 stable chronic patients with schizophrenia and found no cognitive effect. McGuire et al. [16] added 1000 mg CBD/day versus placebo in 88 patients with schizophrenia and demonstrated a trend toward cognitive improvements in chronic patients with schizophrenia. Bhattacharyya et al. [36] examined brain response to a single dose of CBD (600 mg) or placebo during a verbal learning fMRI task in individuals meeting the criteria for APS and found that the CBD group had greater activation in the right caudate, parahippocampal gyrus, and midbrain compared to the placebo group, providing some preliminary experimental medicine data to support potential improvement in cognitive function with CBD in this population.

## CBD, neurohormones, and inflammation

The higher incidence of schizophrenia in urban areas, being a part of an ethnic minority, and migrant status may all be related to stress or social defeat and lack of social support [37, 38]. Increased HPA activity is associated with psychotic disorders and may increase the activity of dopamine pathways, contributing to exacerbation of symptoms [39–41]. Other recent advances in the understanding of the biological processes mediating stress have implicated the role of the hypothalamic–pituitary–adrenal (HPA) axis [42] as well as neuroinflammation [43] as important targets for intervention. Elevated serum levels of pro-inflammatory cytokines and chemokines as well as cortisol have been reported in first-episode patients with schizophrenia [44–47] as well as APS [48]. Neurochemical changes associated with neuroinflammation have also been associated with the symptoms of schizophrenia [49]. Translational studies in animal models have shown that stress can mediate changes in the gene expression during key developmental periods via epigenetic mechanisms [38]. For example, chronic psychosocial stress (e.g., defeat stress) alters the gene expression, particularly of brain-derived neurotrophic factor (BDNF), via a range of epigenetic mechanisms. Importantly, epigenetic moderation of BDNF transcription has been shown to be involved in neuroplasticity, suggesting the potential for pre-emptive intervention in early psychosis.

Several studies have examined the role of CBD in stress pathways as well as in anti-inflammatory signaling [50]. Elevated serum levels of pro-inflammatory cytokines and chemokines as well as elevated salivary cortisol have been reported in APS, first episode, and never-medicated patients, with schizophrenia, and thus may play a role in neuropathological changes in the early stages of illness [44–48]. The administration of CBD in early psychosis is of interest because of the beneficial effect on anxiety [51] and normalization of the biological stress response through serotonin 1A receptor agonism [52]. CBD has been shown to have antioxidative, anti-inflammatory, and neuroprotective effects [53–55]. Cannabinoids can modulate immune reactions in the periphery and brain, influence T-cell balance, cytokine expression, and synchronize balance between neuroinflammation and neurodegeneration processes [56]. CBD has been shown to uniquely target immunometabolism regulation of glia, preventing mitochondrial dysfunction, thus potentially lessening the impact of inflammation and oxidative stress in psychosis [57–61]. In the current study, we will investigate whether factors such as inflammation mediate the response to CBD.

## CBD metabolic and appetite effects

Endocannabinoids also have powerful roles in eating behavior [62, 63], reward [64], and anxiety [65], indicating these neurotransmitters may play a role in reducing hyperphagia and metabolic abnormalities that are present early in the course of psychotic illness [66, 67] and associated with antipsychotic administration. CB1 antagonists have been shown to markedly reduce food intake, significantly reduce weight gain, and regulate serum glucose in mouse models [68]. Although this topic needs further study a recent meta-analysis found that in four of six randomized control trials with CBD (one in schizophrenia, five in epilepsy), in four of 6 randomized controlled trials, CBD was shown to reduce appetite. Although this was reported as an adverse event in these studies, it may be a favorable side effect profile in patients with antipsychotic-induced weight gain [69].

## CBD and neurophysiology

The neurophysiological effects of CBD have been studied in animal models of psychosis [70–74] and in human studies. Prepulse inhibition (PPI) of the startle response, used to assess sensorimotor gating, is deficient in some APS studies [75] and is associated with neurocognition and psychosocial functioning. Peres et al. [76] found that peripubertal treatment with CBD improves PPI deficits in an animal model for schizophrenia. The effects of CBD on PPI have yet to be studied in humans. Event-related responses such as mismatch negativity (MMN) have been shown to be impaired across all stages of psychotic illness [77]. THC administration reduces MMN amplitude in humans, while the use of a cannabis extract containing both THC and CBD enhances the MMN amplitude [78]. By including a neurophysiologic component, we plan to use an experimental medicine design to identify potential markers of treatment response.

## Design and methods

### Aims of the study

The specific aims of this project are to determine if CBD augmentation compared to placebo: significantly modulates psychotic symptoms, cognitive symptoms, pro-inflammatory, and neurohormone biomarker levels, appetite and eating behavior, metabolic measures, and lastly changes neurophysiological abnormalities.

It is hypothesized that CBD will reduce symptoms, improve neurocognition, reduce markers of inflammation, reduce harmful eating behaviors, and improve information processing. Importantly, predictors, moderators, and mediators of the CBD effects will be examined. A better understanding of the mechanism by which CBD acts in early psychosis and the identification of which individuals are more likely to respond to CBD can inform treatment development and personalize treatment.

### Overall design

The study will be conducted in the UCSD CARE (University of California, San Diego, Cognitive Assessment and Risk Evaluation) Early Psychosis Program where we have a multidisciplinary team of psychiatrists and psychologists with expertise in early psychosis, integrative medicine techniques, biomarkers, and clinical trials. UCSD CARE early psychosis and treatment program is in the Psychiatry Department of the University of California San Diego, California. The CARE early psychosis clinic sees up to 4 new patients per week, generally consisting of a relatively high percentage of patients who would be eligible for this study. The catchment area is the entirety of the multicultural and diverse county of San Diego. The coordinating team is part of UCSD CARE and is comprised of the PI, a clinical psychologist, a graduate student, a clinical research coordinator, and three research associates. The research associates are responsible for identifying potential recruits via outreach and referral screenings, consenting participants, and implementing the test battery. The clinical psychologist and graduate student are responsible for administering the clinical measures. The testing procedures are overseen by the clinical research coordinator who manages the study database and coordinates the study with the outside collaborators. The collaborators on this project are the two funding agencies. The first agency is the Krupp Center of Integrative Research at UCSD co-directed by the statistician consulting on this project. The second agency is the Center for Medicinal Cannabis Research (CMCR) which acts as the IND sponsor, monitors the study progress, provides ongoing assistance to the PI on study design and any issues that arise, and will provide assistance in analyzing the study results. The CMCR will also host the collected data and provide long-term storage for the biological samples collected during the study. The samples will not undergo genetic analysis and will be used only for metabolic and inflammatory analysis and will not be specifically stored for future use.

**Fig. 1:**
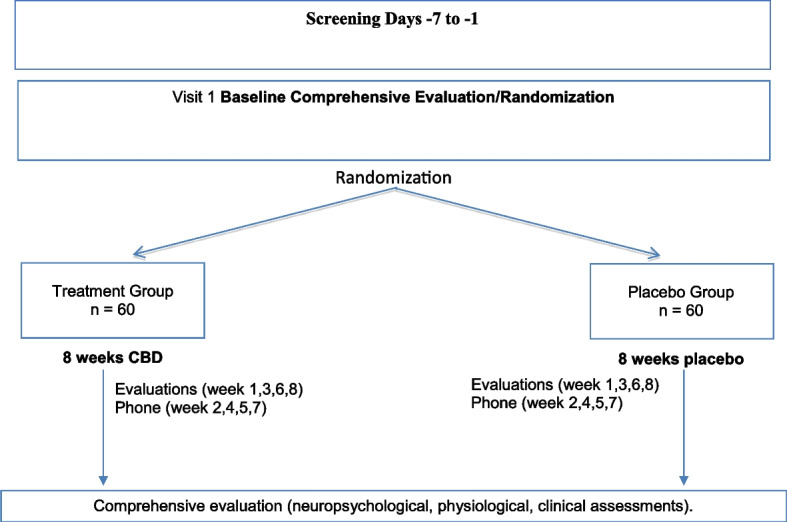
Study design

**Fig. 2:**
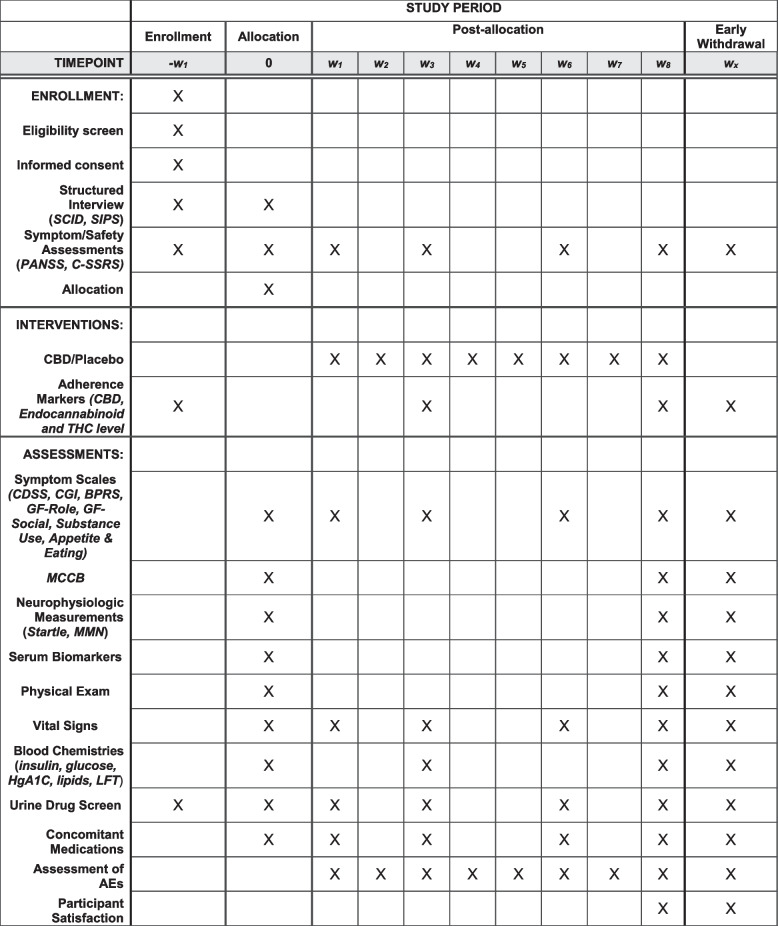
SPIRIT figure

As outlined in the study design (Fig. 1) and SPIRIT (Fig. 2), the trial will include 120 patients in the early stages of psychosis who will be randomly assigned in a 1:1 ratio to receive 1000 mg of cannabidiol or matching placebo as an add-on to current treatment to in a 8-week double-blind superiority randomized controlled trial to be performed over 3 years. The unblinded study statistician has created a list of participant identification numbers with randomized treatment assignments using the SPSS software prior to the start of the study subject recruitment. Blocked randomization will be used with a random block size (4 or 6). Subjects will be randomized to the study treatment group in a 1:1 ratio. No stratification method will be used. A research associate on the team who is not part of the study will be provided with that subject’s randomization code. Any violations of the inclusion and exclusion criteria, reasons for randomization failure including major protocol deviation, lost to follow-up, voluntary withdrawal, and study termination, will be communicated to the study statistician. This research associate is also the only person who is not blinded and who has access to the list that identifies the unique numbers on the relabeled bottles of the two products. They are then responsible for assigning the bottles to the patient. All clinical staff and participants involved in the study will be blinded with the sole exception of the independent statistician. The design is sufficiently powered to answer questions about the efficacy of CBD as an augmentation to antipsychotics in EP, predictors, mediators, and moderators of treatment response and further our understanding of the mechanism of action of CBD in early psychosis. Symptoms, eating behaviors, metabolic parameters, and hormonal and neuroimmune marker levels will be evaluated at baseline, 3 weeks, and 8 weeks, and neurocognitive and neurophysiologic assessment will be performed at baseline and 7 weeks. We will work closely with the UCSD Center for Medicinal Cannabis which has performed multiple CBD trials. UCSD IRB approval, FDA IND approval, and ClinicalTrials.gov registration are in place. Data will be deidentified and stored in RedCAP, a secure dataset available through UCSD. A data monitoring committee is made up of members of both funding agencies, which has assisted in the development of the database and ensuring compatibility with data forms. There is a Data and Safety Monitoring Board (DSMB) that reviews the progress of the study, recruitment, dropouts, and adverse events of trial participants on an annual basis, the board is made up of an independent group of investigators. Any protocol modifications will be reported to IRB, trial registry, and DSMB.

### Participants

In order to be eligible to participate in this study, an individual must meet all of the following criteria:

1. Between the ages of 16 and 30.
2. Acceptable diagnoses will include attenuated psychosis syndrome (APS) per the Structured Interview for Prodromal States (SIPS) [79] or first-episode psychosis (onset within the last 2 years) and meet one of the following diagnoses: psychosis NOS, schizophreniform, schizophrenia, and schizoaffective per the Structured Clinical Interview for DSM-V [80].
3. Stabilized on a current treatment regimen for at least 4 weeks prior to initiating the trial consistent with the FDA-NIMH-MATRICS guidelines for clinical trial design for clinical enhancing drugs [81]:
  - Clinically stable and in a nonacute phase of their illness for at least 2 months
  - If currently on antipsychotics, will have been maintained on a current antipsychotic for at least 6 weeks, with no change in antipsychotic dose for the previous 4 weeks
  - Exhibit no more than moderate levels of positive symptoms (defined by ratings of ≤ 4) on PANSS items P1 (delusions), P2 (conceptual disorganization), P3 (hallucinatory behavior), P5 (grandiosity), P6 (suspiciousness), and G8 (unusual thought content), but still symptomatic (defined by ratings ≥ 2 on positive symptoms items); no more than a minimal level of depressive symptoms as assessed by the Calgary Depression Scale for Schizophrenia (CDSS) [82]

An individual who meets any of the following criteria will be excluded from participation in this study:

- Concomitant medical or neurological illness
- Significant head injury
- Current substance misuse
- IQ < 80 (below which is considered at least borderline-impaired on Stanford-Binet Fifth Edtion)
- High suicidal risk assessed by the Columbia-Suicide Severity Rating Scale (C-SSRS) [83]
- Pregnant women and those who do not agree to avoid becoming pregnant
- Patients requiring treatment with azelastine, fluticasone, dronabinol, valproic acid, or divalproex sodium

If clinically significant, untreated symptoms are found to be present at baseline, or are found to develop during the course of the study, participants will be advised to consult with their mental health care provider and will also be provided with referrals to UCSD (e.g., UCSD CARE Early Psychosis Specialty Clinic-clinical arm of the CARE) and appropriate clinicians in the community. These participants will continue to be followed in the study, with information on treatment recorded in the database.

### Informed consent and biospecimens

On the initial screening visit, the purpose of the study will be explained by a research associate. Individuals who are interested in participating in the study will receive more detailed information, both orally and with a written informed consent. Potential study participants will be asked to sign a separate release of information as well as the UCSD HIPAA form in case information needs to be obtained or released to a third party. Consenting will be completed by experienced research staff. In case of minor participants, both assent from the adolescent and consent from the parent/guardian will be completed.

Consent forms will be written in language that is comprehensible to individuals who have at minimum an eighth grade education. The consent form will contain information about the nature of the interviews, cognitive testing, fluid sampling, and neurophysiological procedures. Procedures for maintaining the confidentiality of data will also be described.

To assess the comprehension of the information provided, after reading the consent forms, subjects or parent/guardian will be asked to provide a statement of their understanding of the research, the research procedure, any risks or discomfort involved, possible benefits of the study, and their right to withdraw at any time. If a subject indicates an inability to comprehend the provided material or is judged to be unable to comprehend this material, that individual will not be permitted to participate in the research. Subjects will receive copies of the signed consent documents, HIPAA authorization forms, and “The Experimental Subject’s Bill of Rights.”

For participants who start as minors and become of age (18 years old) during the length of the study, efforts will be made to obtain consents from participants who completed the active phase of the study but whose biospecimen continues to be stored. A waiver of consent is requested with the goal to continue storing previously collected and stored biospecimen in the case that participants who have come of age cannot be reached. The continued storage of the biospecimen is considered a minimal risk to subjects, given that strict confidentiality as well as safety of storage procedures will be maintained as described in extensive documentation approved by our IRB. The rights and the welfare of the participant are not adversely impacted given that study procedures will have been completed at that time point, and none of the results of the research using the biospecimen would affect clinical decisions about the individual’s care because the results are analyzed after the fact. The biospecimen storage and additional analysis of biospecimen cannot be carried out without the waiver. There is no plan for genetic and/or molecular analysis of biomarkers, only routine biomarker quantification, without long-term storage for specific future studies. If a waiver is not obtained, biospecimen will need to be destroyed and valuable opportunity for scientific knowledge that can be derived from it will be lost.

### Treatment

The Epidiolex and matching placebo are manufactured and supplied by Jazz Pharmaceuticals. The Epidiolex formulation is a 100-mg/mL solution. The CBD compound will be dosed at 1000 mg/day administered in two divided doses after an initial 1-week lead-in of 500 mg. The dose of CBD was selected based on previous controlled trials that demonstrate the efficacy of CBD in patients with schizophrenia [16]. The active pharmaceutical ingredient in cannabidiol oral solution is a pharmaceutical-grade synthetic cannabidiol manufactured according to the current good manufacturing practice (cGMP). It is an off-white to pale yellow resin or crystal substance that is soluble in several organic solvents with an acid dissociation constant (pKa) of 9.64. The solution is a clear, colorless to pale yellow to brown colored solution filled into a 30-mL amber glass bottle of 100 mg/mL strength (i.e., 3000 mg per container) that will be stored at a controlled room temperature (20 to 25 °C, 68 to 77 °F) at the study center where the investigational product will be dispensed. DEA regulations are followed and detail specific security requirements for storage of the investigational product in a “securely locked, substantially constructed cabinet.” Investigators must notify the DEA of the theft or significant loss of any controlled substances within 1 business day of discovering such loss or theft. Any loss in investigational products must be reported to the sponsor. The matching placebo is identical in texture, color, taste, and packaging and will be administered in the same volume as the CBD, so that parents, participants, and investigators should not be able to detect differences between the two treatments. Upon reception of the Epidiolex and placebo bottles, they are all relabeled with an identical label and each assigned a unique number by a team member who is not part of the study and who will be the only person with access to the list identifying the numbers with the vial contents.

Adherence will be assessed during a weekly check-in, bottle inspection, and CBD blood levels at the beginning, 3 weeks, and end of the trial. Participants will be queried as to whether they have missed doses of antipsychotic or study medication. Ease of study recruitment and attrition from the study will also be documented.

Confidentiality will be maintained by assigning each subject a study number and coding all data collected with that number. Identifying information will not be stored on computer databases and will not be stored with the study subject number. All computer databases are password-protected, and hard copies of all data and records will be stored in locked filing cabinets. All study personnel will be certified to conduct research with human subjects and will be aware of the importance of maintaining strict confidentiality.

### Assessments

#### Clinical battery

All items in the clinical battery are administered by laboratory personnel. The Structured Clinical Interview for DSM-V (SCID-I) [84] will be used to assess axis I psychotic disorders and confirm eligibility for the clinical trial. The Structured Interview for the Attenuated Psychosis Syndrome (SIPS) [79] will be used to determine APS eligibility for the study. Symptoms will be assessed with the PANSS [85], C-SSRS [83], and CDSS [82]. Relapse will be defined as any one of the following: psychiatric hospitalization for psychosis, an increase of 25% from baseline in the total PANSS, deliberate self-injury, suicidal or homicidal ideation, or violent behavior. The Systematic Assessment for Treatment Emergent Events (SAFTEE) [86] will be used to assess side effects during the course of the study. The neurocognitive assessment battery derives mainly from the MATRICS Consensus Cognitive Battery (MCCB) [87] which has excellent psychometric properties, utility as a repeated measure, and relates to functional outcome. The following domains are included in the MCCB: (1) estimated IQ—Wechsler Adult Intelligence Scale Vocabulary and Block Design [88]; (2) learning and memory—Hopkins Verbal Learning Test [89, 90] and Brief Visual Memory Test–R [91]; (3) processing speed—Brief Assessment of Cognition in Schizophrenia: Symbol Coding [92, 93], Category Fluency [94], and Trail Making Part A [95]; (4) attention/vigilance—Continuous Performance Task Identical Pairs [96]; (5) working memory—WMS III Spatial Span [97] and Letter-Number Span [98]; (6) executive functioning—Neuropsychological Assessment Battery Mazes [99] and Wisconsin Card Sorting Test [100]. The MCCB has multiple versions of the tests available for repeated assessment. Within each domain, standardized scores are obtained and then combined into a single score per established methods [101]. A Global Cognitive Index is then generated by combining all domain scores.

#### Inflammatory and neurohormone biomarker assays

We have selected a panel of biomarkers based on our pilot study of neuroinflammation in early psychosis funded by an R21 from NIMH. Biomarkers will be assayed in the UCSD Clinical Research Biomarker Laboratory of Cris Achim MD, PhD. Morning blood samples will be drawn and following centrifugation and separation, and plasma or serum will be stored at − 80 °C until assays are conducted. Levels of inflammatory biomarkers will determined in duplicate using mesoscale assays for biomarkers with internal controls (Meso Scale Discovery, Rockville, MD). The biomarkers include pro-inflammatory cytokines, chemokines, markers of vascular injury (C-reactive protein [102], vascular endothelial growth factor), and neural plasticity (BDNF).

We will utilize procedures for saliva collection and cortisol assay used in our previous studies [47]. Three saliva samples will be obtained at hourly intervals in the morning of each assessment. Sample numbers and collection time will be noted and stored at − 20 °C until assay. Participants will be given instructions regarding food and activity restrictions. For the salivary cortisol assay, the Salimetrics (Salimetrics, LLC, College Park, PA) High Sensitivity Salivary Cortisol Enzyme Immunoassay Kit is used. This assay captures the full range of salivary cortisol levels (0.003 to 3.0 μg/dL) requiring only 25 µL of saliva per test. Samples are assayed in duplicate.

#### Eating behavior and metabolic profile

Eating behavior will be assessed with the Dutch Eating Behaviors Questionnaire, Three-Factor Eating Questionnaire, and Food Diary. The metabolic profile will be assessed with anthropomorphic measurements (height, weight, basic metabolic index [BMI]) and plasma metabolic measures (insulin, glucose, HgA1C, lipids).

#### Neurophysiological methods

Neurophysiological markers can provide corroborative data for treatment-related changes in underlying brain chemistry and associated changes in clinical symptoms of psychosis. The following evaluations will be conducted at baseline and at the end of the study.

In the startle prepulse inhibition (PPI) paradigm [103], electrodes (Ag/AgCl) are placed below and at the outer canthus of each eye. A customized startle-stimulus generating program (Grace Design Model m902 Amplifier and Neurobehavioral Systems Presentation software) will be used. The startle stimuli will be presented binaurally through headphones (TDH-39P) and include 70 dB [A] broadband background noise with a pulse (115 dB [A], 40-ms noise burst) presented either alone or following (30, 60, or 120 ms) a prepulse (86 dB [A], 20-ms noise burst). EMG activity will be analyzed using Brain Vision Analyzer (Cortech Solutions, Wilmington, NC) and high-pass filtered at 28 Hz at 12 dB/Oct. The waveform is then smoothed using a 40-Hz 24 dB/Oct low-pass filter. The magnitude of the peak startle response (highest point between 30 and 100 ms) is determined. Subjects with a relative lack of startle-elicited eye blink are excluded. The outcome is PPI or the percentage of change in startle magnitude to prepulse + pulse versus pulse-alone trials.

Stimulation, recording, and analysis techniques for calculating MMN amplitude will follow established methods [104, 105]. Participants will be presented with binaural tones (1 kHz computer-generated square wave stimuli, 85 dB[A] SPL, 1 ms rise/fall) with a fixed stimulus onset-to-onset asynchrony of 500 ms. Standard (*P* = 0.90; 50 ms duration) and deviant (*P* = 0.10; 100 ms duration) tones will be presented to participants in pseudorandom order while they watch a silent video. MMN waveforms will be generated by subtracting event-related potential (ERP) waveforms in response to standard tones from the ERPs generated in response to the deviant tones. The MMN amplitude will be measured as the mean voltage from 135 to 205 ms. The primary dependent measure is MMN amplitude at electrode Fz.

#### Safety and other assessments

All participants will receive frequent health check-in appointments as part of the study. These will occur at baseline, after 1 week after initiating treatment, and then at 4 and 8 weeks. Assessments included in this visit are intended to screen for adverse events that have been described in past studies of CBD notably sedation and GI effects as above, as well as any other adverse events not previously reported. Vital signs will provide information about any possible elevations in temperature, or changes in heart rate or blood pressure. Blood samples will be collected to ensure that there are no adverse effects of the drug in terms of renal and hepatic functioning. In the case that any adverse event is reported, the investigator will assess its severity and relationship to the drug. They will determine how to proceed with treatment. It may include stopping treatment altogether if that is warranted. Detailed documentation will be kept of any and all adverse events. All participants will be given contact information to reach the principal investigator and study staff. This will include a number that can be reached 24/7 in case of emergency. It is possible that early psychosis patients will develop worsening symptoms and require emergency treatment or a higher level of care. In these situations, we will offer alternate treatments that will be consistent with the standard of care in the community. Individuals will be referred to the appropriate resource based on their insurance funding and acuity of the problem. Discontinuation from study intervention may occur if the patient is noncompliant with the treatment or fails multiple (more than 3) follow-up appointments and does not reschedule. Participant satisfaction will be assessed with an exit survey regarding the overall satisfaction with the trial including the tolerability.

### Data analysis

Power analyses were conducted allowing for a 20% dropout rate, and a sample size of 120 early psychosis patients (60 per group), measured at 3 time points, was calculated as sufficient to detect a treatment difference in PANSS/CGI total score, biomarker assessments, food intake and metabolic indices, and neurophysiological measures between CBD and placebo on the change from baseline to end of treatment, with a two-tailed 1% significance level and 80% power. We selected a sample size that provided a minimum of 80% power for medium effect sizes based on the anti-inflammatory outcome measures reported in multiple studies [106–108] that show medium effect sizes (Cohen *d* = 0.47; Hedges *g* =  − 0.39 in EP) for clinical symptoms.

Ensuring adequate recruitment requires a number of strategies, including providing announcements to local primary care practitioners, including community pediatricians, family practice physicians, educators, clinical social workers, psychologists, and psychiatrists. Ads are also posted on various social media platforms. Additionally, ResearchMatch.org and clinicaltrials.org will be utilized as a recruitment tool for this protocol. We estimate that we will be able to recruit five new patients every month based on past referrals.

Data will be analyzed using a mixed model [109] for all clinical, neurocognitive, biomarker, eating behavior, and electrophysiological markers. This method allows the inclusion of subjects with missing data or those who were terminated early in the study, without relying on data imputation procedures. This method provides an estimate of the individual variability around the population trend, the variability of the individual intercepts (baseline values) and slopes (changes across time), and the correlation between them. The model will include a random intercept, a random effect for assessment time, and fixed effects for comparison groups and group-by-time interaction. A fully saturated treatment-by-time model will be utilized for inference. Co-variance structure will be chosen based on Akaikes Information Criterion (AIC). Random group-level treatment effects will also be evaluated for importance based on the model AIC. Any group-level effects to be incorporated into the model. Denominator degree’s of freedom will be calculated using the Kenward-Roger small sample correction. Data will be analyzed from all randomized subjects on whom we have a baseline assessment and at least one post-baseline evaluation. Hypothesis 5 will be testing using the multiple regression procedure. All tests will be done using the SPSS version 24.

We will explore predictors (moderators) of treatment response including baseline symptom severity, neurocognition, biomarker (cortisol and inflammatory markers), and neurophysiological markers by building hierarchical linear models (HLM). The independent variables are the treatment group, moderator, and the treatment × moderator interaction. Mediation analyses will use a similar linear mixed models approach. Two conditions must be met: (1) correlation between the mediator and treatment and (2) relationship between the mediator and outcome. First, we will test the effect of the treatment group and the group × time interaction on the mediator, and we expect a statistically significant group × time interaction. Second, we will test the effects of mediator by group interaction on the outcome in the model that includes group and time; we expect a statistically significant mediator × time or mediator × group × time interaction.

Number, time, reasons, and grade (severity) of adverse events will be summarized overall and by treatment using mean (SD) or median (IQR), range, and *N* (%) as appropriate. The proportion of subjects with adverse events will be compared between treatments using the McNemar test. The number of adverse events will be compared between treatments using the Wilcoxon signed-rank test. Serious adverse events are reported to the IRB within 24 h of the occurrence of the event and follow the proper procedures regarding the reporting of expectedness, seriousness, severity, and causality. The study subjects are instructed to report any new unusual symptoms, suspected side effect, or health-related events as soon as they occur during the 7-week trial period. On the weeks where they do not have an in-person visit, the subjects are contacted by phone by the clinical psychologist and asked a series of questions to assess any side effects that they might be experiencing. All serious adverse events are also reported in the annual DSMB report.

Baseline demographic measures and relevant diagnostic characteristics will be summarized as described under general approach. If dropout occurs, the characteristics of participants who withdraw from the study prematurely will be compared to those who complete the study as described under general approach. We used the SPIRIT reporting guidelines for this manuscript [110].

## Discussion

The proposed study will be the first to examine the effects of CBD in early psychosis in a randomized control trial. It is hypothesized that CBD will improve symptoms, neurocognition, markers of stress and inflammation, eating behaviors, and neurophysiological measures. Importantly, moderators and mediators of the CBD effects will be explored. Potential moderators of response include baseline symptom severity, neurocognitive deficits, cortisol, and inflammatory biomarkers. We will explore improvements in inflammatory biomarkers as mediators of improvement in clinical symptoms, neurocognition, and metabolic profile. If a reduction in inflammatory measures mediates improvement in outcome, this would suggest that individuals with evidence of inflammation would benefit most from the proposed interventions. Finally, this will be the first human study to explore the effects of CBD on neurophysiological markers such as PPI and to examine whether a change in either MMN or PPI is a predictor of response to CBD in other domains. A better understanding of which early psychosis individuals are more likely to respond to CBD can inform treatment planning, specifying treatment for a particular individual based on presenting complaints or biomarker profile. A better understanding of which individuals are more likely to respond to CBD can inform treatment planning and personalize treatment.

## Trial status

This trial is under IRB Project #200,789, version 17 of the University of California, San Diego Human Research Protections Program. Recruitment began on March 15, 2022, with an expected end date of October 1, 2024.

## Data Availability

The final access to the trial dataset will be available to funding agencies. In addition to the CARE team members who are involved in the data collection and analysis, the trial dataset will be shared with the CMCR and the Krupp Center of Integrative Research. Data collected for this study will be analyzed and stored at the CMCR. The de-identified, archived data may be used by other researchers including those outside of the study upon approval by CMCR and with appropriate regulatory approval. Data and results will be sent for publication on trial and manuscript completion. The consent material is included as supplementary information.

## Abbreviations

AE: Adverse event
AIC: Akaikes Information Criterion
APS: Attenuated psychosis syndrome
BDNF: Brain-derived neurotrophic factor
CARE: Cognitive Assessment and Risk Evaluation
CBD: Cannabidiol
cGMP: Good Manufacturing Practice
ECB: Endocannabinoid
ERP: Event-related potential
HPA: Hypothalamic-pituitary-adrenal
MCCB: MATRICS Consensus Cognitive Battery
MMN: Mismatch negativity
PANSS: Positive and Negative Syndrome Scale
PFC: Prefrontal cortex
PPI: Prepulse inhibition
SAFTEE: Systematic Assessment for Treatment Emergent Events
SCID-I: Structured Clinical Interview for DSM-V
THC: Tetrahydrocannabinol
UCSD: University of California, San Diego
